# Appeal of Tobacco Quitline Services Among Low-Income Smokers

**DOI:** 10.5888/pcd20.220214

**Published:** 2023-03-02

**Authors:** Lauren M. Grimes, Rachel Garg, Olivia Weng, Jennifer M. Wolff, Amy McQueen, Kelly M. Carpenter, Matthew W. Kreuter

**Affiliations:** 1 Health Communication Research Laboratory, Brown School at Washington University in St Louis, St Louis, Missouri; 2 Division of General Medical Sciences, School of Medicine, Washington University in St Louis, St Louis, Missouri; 3 RVO Health, Center for Wellbeing Research, Seattle, Washington

## Abstract

**Introduction:**

State tobacco quitlines are delivering cessation assistance through an increasingly diverse range of channels. However, offerings vary from state to state, many smokers are unaware of what is available, and it is not yet clear how much demand exists for different types of assistance. In particular, the demand for online and digital cessation interventions among low-income smokers, who bear a disproportionate burden of tobacco-related disease, is not well understood.

**Methods:**

We examined interest in using 13 tobacco quitline services in a racially diverse sample of 1,605 low-income smokers in 9 states who had called a 2-1-1 helpline and participated in an ongoing intervention trial from June 2020 through September 2022. We classified services as standard (used by ≥90% of state quitlines [eg, calls from a quit coach, nicotine replacement therapy, printed cessation booklets]) or nonstandard (mobile app, personalized web, personalized text, online chat with quit coach).

**Results:**

Interest in nonstandard services was high. Half or more of the sample reported being very or somewhat interested in a mobile app (65%), a personalized web program (59%), or chatting online with quit coaches (49%) to help them quit. In multivariable regression analyses, younger smokers were more interested than older smokers in digital and online cessation services, as were women and smokers with greater nicotine dependence.

**Conclusion:**

On average, participants were very interested in at least 3 different cessation services, suggesting that bundled or combination interventions might be designed to appeal to different groups of low-income smokers. Findings provide some initial hints about potential subgroups and the services they might use in a rapidly changing landscape of behavioral interventions for smoking cessation.

**Summary**

**What is already known on this topic?**

State tobacco quitlines have expanded the types of cessation assistance they offer.

**What is added by this report?**

This study describes the level of interest in 13 cessation services among low-income smokers in 9 states. Interest varied across subgroups.

**What are the implications for public health practice?**

Matching cessation services or bundles of services to groups of low-income smokers may increase their engagement in quitline use and smoking cessation.

## Introduction

Telephone counseling for smoking cessation is an evidence-based intervention (1) that has long been the centerpiece of tobacco quitline services (2). However, state tobacco quitlines have been expanding the types of cessation assistance they provide beyond telephone counseling. From 2008 to 2021, the proportion of state and territorial quitlines that offered online self-help tools increased from 42% to 94%, automated email messaging increased from 30% to 79%, instant messaging or emailing with a cessation counselor increased from 30% to 64%, and online chat rooms with other smokers increased from 29% to 66% (3,4). Tracking of quitlines offering text messaging began in 2009; the percentage of quitlines offering text messaging increased from 2% in 2009 to 85% in 2021(3,4). Yet awareness of these newer services is low, and both smokers and nonsmokers primarily think of quitlines as providing telephone-based services (5).

Studies of other health behavior interventions suggest that younger adults, people with greater computer proficiency, and people living in rural areas have greater interest in text, mobile telephone, and internet interventions, whereas older adults tend to be less interested in mobile telephone interventions and more interested in printed materials (6–8). Because smokers living below the federal poverty level tend to smoke at higher rates than other groups and bear a disproportionate burden of tobacco-related disease (9), understanding their preferences in a changing landscape of behavioral health interventions is both timely and important.

Quitline services are free and available in every state, making them accessible for low-income smokers who lack the means to access other types of cessation assistance (4). Quitline use has declined substantially since the COVID-19 pandemic (10). Gaining insights into smokers’ interest in and demand for different types of cessation assistance could help quitlines refine and target their offerings to reinvigorate usage.

This study examined interest in 13 current or potential quitline services among a large sample of low-income adult daily smokers in 9 states. We examined the overall level of interest in each service and explored differences in interest by participant characteristics. We also examined the level of interest by type of service, such as technology-mediated cessation assistance and assistance delivered interpersonally. We hypothesized that services delivered through available technology, such as web-based services, may be more popular among younger smokers, while older smokers may prefer interpersonal options, such as telephone-based services.

## Methods

All research procedures were approved by the Washington University Institutional Review Board (no. 202002125).

### Sample

We analyzed baseline data from an ongoing intervention trial testing 2 approaches to increase smoking cessation among low-income smokers. Participants were recruited after calling 2-1-1 helplines in Connecticut, Indiana, Louisiana, Missouri, North Carolina, New Mexico, South Carolina, Tennessee, and Washington. 2-1-1 helplines are not tobacco quitlines; rather, they provide callers with information and referrals to local service agencies for assistance with social needs such as food or housing (11). We did not establish an income cutoff for inclusion in the study, but callers to 2-1-1 helplines overwhelmingly have a low income (11). For example, in the last 2 large studies we conducted among 2-1-1 participants, more than 80% of participants in each study reported having less than $20,000 in annual pretax household income (12,13). After receiving standard service from 2-1-1 operators, callers were asked about their smoking status and, for adult daily smokers, their interest in participating in a study for smokers. University research team members then contacted those who expressed interest and agreed to share their contact information. Those who were reached, met eligibility criteria (aged ≥21 years, daily smoker, no rules banning smoking in their home, not pregnant, speaks English), and provided informed consent were administered a baseline telephone interview. Participants who completed the baseline survey were mailed a $25 grocery store gift card.

Of 6,338 callers who agreed to share their contact information from June 1, 2020, through September 6, 2022, 3,379 (53%) were reached by the study team during that period. Of those reached, 1,170 (35%) were not interested in participating, and 604 (18%) did not meet eligibility criteria. A total of 1,605 smokers met all eligibility criteria and completed the baseline interview.

### Dependent variables

**Interest in quitline services.** We identified 13 types of cessation assistance that were available through at least 1 state tobacco quitline as of March 2020: 1) nicotine patches, gum, or lozenges; 2) a toll-free telephone number smokers could call at any time; 3) a printed quit smoking booklet; 4) online lessons and exercises; 5) a specially trained quit coach to call smokers on the telephone; 6) personalized email messages; 7) being able to text or chat online with a quit smoking coach; 8) a mobile app for smokers to track their cravings, triggers, and smoking patterns with motivational messages and tips; 9) a personalized web program to track progress toward cessation; 10) personalized text messages; 11) a chat group online with others who have quit smoking or are trying to quit; 12) being able to a talk with a quit smoking coach in person; and 13) sending information to smokers’ family and friends to learn how to help them quit. For each service, we asked participants to “tell me how interested you would be in receiving that service if you were thinking about quitting.” Participants were informed that all of the services were currently available in some states and told to assume that all services would be free of charge. Response options were “very interested,” “somewhat interested,” and “not interested.”

We tallied the number of states that offered each quitline service in 2021 by using the North American Quitline Consortium map and classified services as standard quitline services if they are offered by 90% or more of US quitlines and nonstandard if they are offered by less than 90% of quitlines (4). Of the 13 types of services examined, the following were considered standard: 1) nicotine patches, gum, or lozenges; 2) a toll-free telephone number smokers could call at any time; 3) a printed quit smoking booklet; 4) online lessons and exercises; and 5) a specially trained quit coach to call smokers on the telephone. All others were classified as nonstandard. To guard against potential social desirability response bias, we predicted the proportion of participants who indicated being very interested in each quitline service. We also summed the number of quitline services that each participant was very interested in receiving (range 0–13) and calculated sample means for this variable.

### Independent variables

We examined potential correlates of being very interested in a quitline service, including psychosocial, tobacco-related, and demographic characteristics of participants.

Perceived stress was measured by using the Cohen 4-item Perceived Stress Scale (PSS-4), which measures how frequently life situations in the past month were perceived as stressful (14). We calculated summary scores ranging from 0 to 16, with higher scores indicating greater perceived stress (Cronbach α = .58).

Depressive symptoms were measured by using the 2-item Patient Health Questionnaire-2 (PHQ-2) depression screener, which measures the frequency of depressive mood and anhedonia over the past 2 weeks (15). We calculated summary scores ranging from 0 to 6, with higher scores indicating greater frequency of depressive symptoms and a score of 3 or higher indicating possible depression.

Social support was measured by using 4 items adapted from the Patient-Reported Outcomes Measurement Information System (PROMIS) emotional support measure (16). Participants rated how much they agree or disagree (strongly disagree/disagree/agree/strongly agree) that they 1) have someone who understands their problems, 2) have someone who will listen to them when they need to talk, 3) have someone to turn to for suggestions about how to deal with a problem, and 4) have someone who will help them if they decide to quit smoking. We calculated mean scores ranging from 1 to 4, with higher scores indicating greater social support (Cronbach α = .84).

Nicotine dependence was measured by using the 2-item Heaviness of Smoking Index (17), which assesses number of cigarettes smoked per day and time between waking up and smoking a first cigarette. Scores range from 0 to 6, with higher scores indicating greater nicotine dependence.

Ambivalence about smoking was measured by using 3 items adapted from an attitudinal ambivalence scale, which assesses conflicting thoughts and feelings about smoking (18). Participants rated how much they agreed or disagreed that they 1) sometimes think smoking is good, while at other times think smoking is bad, 2) feel torn between wanting to smoke and not wanting to smoke, and 3) have mixed feelings about smoking. The response options for these 3 items were strongly disagree (score of 1), disagree (score of 2), agree (score of 3), and strongly agree (score of 4). We calculated mean scores ranging from 1 to 4, with higher scores indicating greater ambivalence about smoking (Cronbach α = .58).

Stage of readiness to quit was assessed by asking whether participants 1) were seriously thinking about quitting smoking in the next 6 months, and if so whether they 2) had a specific plan to quit smoking in the next 30 days (19). Participants not thinking about quitting smoking in the next 6 months were classified as being in the precontemplation stage; participants thinking about quitting in the next 6 months, but without a specific plan, were classified as being in the contemplation stage; and participants thinking about quitting in the next 6 months and having a specific plan to quit in the next 30 days were classified as being in the preparation stage.

Other tobacco-related items assessed whether participants lived with other smokers (yes/no) and used any cessation aid such as nicotine replacement therapy (NRT), Chantix, or “Zyban, bupropion, or Wellbutrin” during past quit attempts (yes/no).

We assessed participants’ age, sex, race, ethnicity, annual pretax household income, highest level of education, whether they had children younger than 18 years living in the home, and whether they lived in a rural or nonrural area, as determined by zip code (20).

### Statistical analyses

All data were managed and analyzed by using R version 3.6.1 (R Foundation for Statistical Computing). We calculated descriptive statistics for all study variables. We used bivariate logistic regression analyses to examine associations between being very interested in each quitline service and each potential covariate. Factors that were associated with interest in any of the 13 quitline services at the α = .05 level in bivariate analyses were included in all multivariable logistic regression analyses. We estimated 13 separate multivariable logistic regression models (one for each quitline service) to identify factors associated with each service by using a common set of independent variables selected on the basis of results of the bivariate analyses. We calculated adjusted odds ratios (AORs) and 95% CIs. Missing data were handled with listwise deletion.

## Results

Most participants were women (68%) and White (47%) or Black or African American (41%). The mean (SD) age was 50.8 (12.0) years. One in 4 participants reported less than a high school education (27%), and 38% reported less than $10,000 in pretax household income per year (Table 1). On average, participants smoked 15.4 (SD, 9.4) cigarettes per day.

**Table 1:** Demographics Characteristics, Psychosocial Factors, and Tobacco Use Among a Sample of Low-Income Adult Daily Smokers in 9 States (N = 1,605) Participating in a Telephone Survey on Quitline Services, 2020–2022^a^

Characteristic	No. (%)^b^
**Demographic**	
**Age, mean (SD), y**	50.8 (12.0)
**Age group, y**	
≤40	361 (23)
41–60	850 (53)
≥61	383 (24)
**Sex**	
Female	1,083 (68)
Male	312 (32)
**Race**	
Black or African American	652 (41)
White	747 (47)
Other^c^	188 (12)
**Hispanic ethnicity**	73 (5)
**Annual pretax household income, $**	
<10,000	579 (38)
10,000–19,999	499 (33)
≥20,000	437 (29)
**Education**	
Less than high school	438 (27)
High school/GED	545 (34)
More than high school	622 (39)
**Children aged <18 y living in home**	406 (25)
**Reside in rural zip code**	249 (16)
**Psychosocial factors, mean (SD)**	
Perceived stress (scale 0–16)	7.6 (3.4)
Depressive symptoms (scale 0–6)	2.6 (1.8)
Social support (scale 1–4)	3.0 (0.8)
**Tobacco-related factors**	
Nicotine dependence (scale 0–6), mean (SD)	3.0 (1.4)
Past use of cessation aids	874 (55)
Live with other smokers	675 (42)
Ambivalence about smoking (scale 1–4), mean (SD)	3.0 (0.7)
Stage of readiness to quit	
Precontemplation	302 (20)
Contemplation	903 (58)
Preparation	345 (22)

Abbreviation: GED, General Educational Development.

a Nine states are Connecticut, Indiana, Louisiana, Missouri, North Carolina, New Mexico, South Carolina, Tennessee, and Washington.

b Values are number (percentage) unless otherwise indicated. Not all categories add to 1,605 because not all participants answered all questions; percentages are based on the number of participants who answered the question. Percentages may not sum to 100% because of rounding.

c Includes American Indian/Alaska Native, Asian/Asian American, Native Hawaiian/Pacific Islander, other, multiple races.

Participants reported high levels of social support, averaging 3.0 on a scale of 1 to 4. The average score for perceived stress was 7.6 (on a 0–16 scale) and for depressive symptoms was 2.6 (on a 0–6 scale) (Table 1).

Most participants (58%) were in the contemplation stage for quitting smoking, and nearly one-quarter (22%) were in the preparation stage. More than half (55%) reported using cessation aids during past quit attempts. Ambivalence about smoking was high (3.0 on a 1–4 scale), and 42% reported living with other smokers (Table 1).

### Interest in quitline services

Among standard quitline services, participants were most interested in receiving NRT (42% were very interested), having a toll-free number they could call at any time (33% were very interested), and receiving a printed quit smoking booklet (27% were very interested). Among nonstandard quitline services, participants were most interested in a mobile app to help them quit (34% were very interested), a personalized web program (27% were very interested), or personalized text messages (22% were very interested). Among both standard and nonstandard services, interest was lowest for receiving calls from a quit coach (17% were very interested), personalized emails (17% were very interested), and sending information to family or friends to learn about helping them quit (14% were very interested). On average, participants reported being very interested in 3 of the 13 quitline services (mean [SD] = 3.1 [3.5] services (Table 2); median [interquartile range] = 2.0 [2.0–5.0]).

**Table 2:** Level of Interest in Standard and Nonstandard Quitline Services Among a Sample of Low-Income Adult Daily Smokers in 9 States (N = 1,605) Participating in a Telephone Survey on Quitline Services, 2020–2022^a^

Quitline service	No. (%)^b^		
	Very interested	Somewhat interested	Not interested
**Standard service**			
Nicotine patches, gum, or lozenge (NRT)	667 (42)	464 (29)	467 (29)
Toll-free telephone number to quitline	533 (33)	511 (32)	558 (35)
Printed quit-smoking booklet	425 (27)	501 (31)	674 (42)
Online lessons and exercises	358 (22)	528 (33)	715 (45)
Telephone calls from quit coach	274 (17)	494 (31)	829 (52)
**Nonstandard service**			
Mobile app to help quit	542 (34)	498 (31)	560 (35)
Personalized web program	425 (27)	515 (32)	657 (41)
Personalized text messages	349 (22)	392 (24)	859 (54)
Chat online with quit coach	320 (20)	460 (29)	817 (51)
Meet quit coach in person	312 (20)	399 (25)	885 (55)
Group chat online with smokers	283 (18)	454 (28)	864 (54)
Personalized emails	268 (17)	367 (23)	963 (60)
Send information to family/friends	225 (14)	301 (19)	1077 (67)
Total number services (0–13), mean (SD)	3.1 (3.5)	3.7 (3.0)	6.2 (4.2)

Abbreviation: NRT, nicotine replacement therapy.

a Nine states are Connecticut, Indiana, Louisiana, Missouri, North Carolina, New Mexico, South Carolina, Tennessee, and Washington.

b Values are number (percentage) unless otherwise indicated. Responses coded as missing (eg, not sure, refused) were excluded, so row totals may not sum to 1,605.

### Associations between participant characteristics and interest in services

In bivariate analyses, Hispanic ethnicity and living with other smokers were not significantly associated with being very interested in any of the quitline services (Supplemental Table 1, Table 2A, and Table 2B in Appendix). All other psychosocial, tobacco-related, and demographic characteristics were significantly associated with being very interested in at least 1 service and were, therefore, included in multivariable models.

Interest in types of services differed by age group. Compared with smokers aged 40 years or younger, smokers aged 41 to 60 years had higher odds of interest in having a toll-free telephone number to call at any time (AOR, 1.47; 95% CI, 1.06–2.04) or a printed quit smoking booklet (AOR, 1.67; 95% CI, 1.17–2.41). Smokers aged 61 years or older had nearly twice the odds of interest in a printed quit smoking booklet compared with smokers aged 40 years or younger (AOR, 1.97; 95% CI, 1.30–3.01) (Table 3). Smokers aged 41 to 60 years also had lower odds of interest in a mobile app (AOR, 0.65; 95% CI, 0.48–0.89), while smokers aged 61 years or older had approximately half the odds of interest in a mobile app (AOR, 0.51; 95% CI, 0.35–0.75) or a personalized web program (AOR, 0.49; 95% CI, 0.32–0.74) compared with smokers aged 40 years or younger (Table 4A).

**Table 3:** Multivariable Logistic Regression Results for Models Examining Associations Between Participant Characteristics and Interest in Standard Quitline Services Among a Sample of Low-Income Adult Daily Smokers in 9 States (N = 1,605) Participating in a Telephone Survey on Quitline Services, 2020–2022

Independent variable	Adjusted odds ratio (95% CI)				
	Nicotine patches, gum, or lozenges (n = 1,340)	Toll-free telephone number to quitline (n = 1,344)	Printed quit smoking booklet (n = 1,344)	Online lessons and exercises (n = 1,344)	Telephone calls from quit coach (n = 1,340)
**Demographic**					
**Age group, y**					
≤40	1 [Reference]	1 [Reference]	1 [Reference]	1 [Reference]	1 [Reference]
41–60	1.15 (0.85–1.55)	1.47 (1.06–2.04)^a^	1.67 (1.17–2.41)^a^	1.02 (0.71–1.46)	1.33 (0.88–2.03)
≥61	0.88 (0.61–1.28)	1.47 (1.00–2.17)	1.97 (1.30–3.01)^a^	0.92 (0.60–1.42)	1.25 (0.77–2.06)
**Sex**					
Male	1 [Reference]	1 [Reference]	1 [Reference]	1 [Reference]	1 [Reference]
Female	1.11 (0.86–1.44)	1.32 (1.00–1.73)^a^	1.54 (1.15–2.07)^a^	1.54 (1.13–2.11)^a^	1.43 (1.02–2.05)^a^
**Race**					
Black or African American	1.06 (0.81–1.40)	1.44 (1.09–1.93)^a^	2.01 (1.48–2.75)^a^	2.18 (1.58–3.03)^a^	2.20 (1.53–3.17)^a^
White	1 [Reference]	1 [Reference]	1 [Reference]	1 [Reference]	1 [Reference]
Other^b^	0.99 (0.68–1.44)	0.87 (0.58–1.30)	1.96 (1.30–2.95)^a^	1.66 (1.07–2.55)^a^	2.07 (1.29–3.30)^a^
**Annual pretax household income, $**					
<10,000	1 [Reference]	1 [Reference]	1 [Reference]	1 [Reference]	1 [Reference]
10,000–19,999	1.12 (0.85–1.49)	1.01 (0.76–1.35)	1.15 (0.85–1.56)	1.25 (0.91–1.73)	0.95 (0.67–1.36)
≥20,000	0.95 (0.70–1.29)	0.92 (0.66–1.26)	0.96 (0.68–1.35)	1.07 (0.75–1.53)	0.90 (0.60–1.35)
**Education**					
Less than high school	1 [Reference]	1 [Reference]	1 [Reference]	1 [Reference]	1 [Reference]
High school/GED	1.14 (0.84–1.54)	1.30 (0.95–1.78)	0.88 (0.64–1.23)	0.88 (0.62–1.24)	1.04 (0.71–1.53)
More than high school	1.14 (0.85–1.54)	1.22 (0.89–1.67)	0.90 (0.65–1.25)	1.02 (0.73–1.44)	1.07 (0.73–1.57)
**Children aged <18 y living in home**					
No	1 [Reference]	1 [Reference]	1 [Reference]	1 [Reference]	1 [Reference]
Yes	0.99 (0.75–1.31)	0.76 (0.56–1.02)	0.96 (0.70–1.31)	0.79 (0.57–1.10)	0.94 (0.65–1.35)
**Reside in rural zip code**					
No	1 [Reference]	1 [Reference]	1 [Reference]	1 [Reference]	1 [Reference]
Yes	1.06 (0.76–1.47)	1.31 (0.93–1.84)	1.44 (1.00–2.07)^a^	1.32 (0.89–1.93)	0.78 (0.48–1.23)
**Psychosocial factors**					
Perceived stress (scale 0–16)	0.99 (0.95–1.03)	1.01 (0.96–1.05)	0.99 (0.95–1.04)	1.04 (0.99–1.09)	1.02 (0.97–1.08)
Depressive symptoms (scale 0–6)	1.03 (0.96–1.11)	1.07 (0.99–1.16)	1.08 (1.00–1.17)	1.03 (0.94–1.12)	1.03 (0.94–1.13)
Social support (scale 1–4)	0.94 (0.81–1.10)	1.09 (0.93–1.29)	1.38 (1.16–1.66)^a^	1.40 (1.16–1.69)^a^	1.05 (0.86–1.28)
**Tobacco-related factors**					
Nicotine dependence (scale 0–6)	1.09 (1.00–1.19)	1.00 (0.91–1.10)	1.06 (0.96–1.17)	1.11 (1.01–1.23)^a^	1.15 (1.03–1.29)^a^
Past use of cessation aids (vs no use)	1.40 (1.10–1.78)^a^	1.37 (1.07–1.76)^a^	1.19 (0.91–1.55)	1.09 (0.83–1.44)	1.45 (1.06–1.99)^a^
Ambivalence about smoking (scale 1–4)	1.33 (1.11–1.59)^a^	1.47 (1.22–1.78)^a^	1.18 (0.97–1.44)	1.20 (0.98–1.48)	1.30 (1.04–1.65)^a^
Stage of readiness to quit					
Precontemplation	1 [Reference]	1 [Reference]	1 [Reference]	1 [Reference]	1 [Reference]
Contemplation	3.94 (2.75–5.78)^a^	4.49 (2.94–7.13)^a^	3.23 (2.07–5.25)^a^	3.87 (2.34–6.81)^a^	4.77 (2.50–10.33)^a^
Preparation	4.65 (3.08–7.12)^a^	5.84 (3.65–9.63)^a^	5.05 (3.11–8.48)^a^	6.20 (3.61–11.24)^a^	7.95 (4.03–17.58)^a^

Abbreviation: GED, General Educational Development.

a Significant at α = .05; because of rounding, some associations have 95% CIs that appear bounded by 1.00.

b Includes American Indian/Alaska Native, Asian/Asian American, Native Hawaiian/Pacific Islander, other, multiple races.

**Table 4A:** Multivariable Logistic Regression Results for Models Examining Associations Between Participant Characteristics and Interest in Nonstandard Quitline Services Among a Sample of Low-Income Adult Daily Smokers in 9 States (N = 1,605) Participating in a Telephone Survey on Quitline Services, 2020–2022^a^

Independent variables	Adjusted odds ratio (95% CI)			
	Mobile app (n = 1,344)	Personalized web program (n = 1,344)	Personalized text messages (n = 1,343)	Chat online with quit coach (n = 1,341)
**Demographic**				
**Age group, y**				
≤40	1 [Reference]	1 [Reference]	1 [Reference]	1 [Reference]
41–60	0.65 (0.48–0.89)^b^	0.74 (0.54–1.04)	1.19 (0.83–1.72)	1.19 (0.82–1.75)
≥61	0.51 (0.35–0.75)^b^	0.49 (0.32–0.74)^b^	0.96 (0.62–1.49)	1.22 (0.78–1.92)
**Sex**				
Male	1 [Reference]	1 [Reference]	1 [Reference]	1 [Reference]
Female	1.64 (1.25–2.15)^b^	1.56 (1.16–2.10)^b^	1.32 (0.97–1.81)	1.82 (1.31–2.56)^b^
**Race**				
Black or African American	1.35 (1.02–1.80)^b^	1.63 (1.21–2.22)^b^	2.00 (1.45–2.76)^b^	1.78 (1.28–2.49)^b^
White	1 [Reference]	1 [Reference]	1 [Reference]	1 [Reference]
Other^c^	0.85 (0.57–1.26)	1.06 (0.69–1.61)	1.03 (0.64–1.61)	1.06 (0.66–1.69)
**Annual pretax household income, $**				
<10,000	1 [Reference]	1 [Reference]	1 [Reference]	1 [Reference]
10,000–19,999	1.08 (0.81–1.44)	1.29 (0.95–1.76)	0.87 (0.62–1.20)	1.14 (0.81–1.59)
≥20,000	1.25 (0.92–1.71)	1.30 (0.93 −1.83)	0.98 (0.69–1.40)	1.09 (0.75–1.57)
**Education**				
Less than high school	1 [Reference]	1 [Reference]	1 [Reference]	1 [Reference]
High school/GED	0.98 (0.72–1.34)	1.13 (0.81–1.57)	0.72 (0.51–1.02)	0.94 (0.65–1.35)
More than high school	1.19 (0.88–1.62)	1.15 (0.83–1.60)	0.84 (0.60–1.18)	1.04 (0.74–1.50)
**Children aged <18 y living in home**				
No	1 [Reference]	1 [Reference]	1 [Reference]	1 [Reference]
Yes	0.88 (0.66–1.17)	0.73 (0.54–1.00)	0.92 (0.66–1.28)	1.07 (0.76–1.49)
**Reside in rural zip code**				
No	1 [Reference]	1 [Reference]	1 [Reference]	1 [Reference]
Yes	0.91 (0.65–1.27)	0.97 (0.67–1.40)	1.08 (0.73–1.59)	1.21 (0.81–1.79)
**Psychosocial factors**				
Perceived stress (scale 0–16)	1.01 (0.97–1.06)	1.01 (0.96–1.06)	0.99 (0.95–1.04)	1.01 (0.96–1.06)
Depressive symptoms (scale 0–6)	1.08 (1.01–1.17)^b^	1.07 (0.98–1.16)	1.11 (1.02–1.21)^b^	1.07 (0.98–1.17)
Social support (scale 1–4)	1.13 (0.96–1.33)	1.21 (1.02–1.44)^b^	1.34 (1.11–1.62)^b^	1.21 (1.00–1.46)
**Tobacco-related factors**				
Nicotine dependence (scale 0–6)	1.11 (1.02–1.22)^b^	1.13 (1.03–1.25)^b^	1.15 (1.04–1.28)^b^	1.17 (1.05–1.31)^b^
Past use of cessation aids (vs no use)	1.40 (1.09–1.80)^b^	1.33 (1.02–1.74)^b^	1.53 (1.15–2.03)^b^	1.36 (1.02–1.82)^b^
Ambivalence about smoking (scale 1–4)	1.23 (1.03–1.48)^b^	1.20 (0.99–1.47)	1.18 (0.96–1.46)	1.37 (1.10–1.71)^b^
Stage of readiness to quit				
Precontemplation	1 [Reference]	1 [Reference]	1 [Reference]	1 [Reference]
Contemplation	3.42 (2.33–5.14)^b^	5.14 (3.15–8.86)^b^	3.92 (2.37–6.90)^b^	6.12 (3.31–12.66)^b^
Preparation	4.39 (2.84–6.90)^b^	6.67 (3.93–11.91)^b^	5.36 (3.11–9.73)^b^	7.29 (3.79–15.49)^b^

Abbreviation: GED, General Educational Development.

a The following services are considered standard quitline services: nicotine patches, gum, or lozenge; toll-free telephone number to quitline; printed quit smoking booklet; online lessons and exercises; telephone calls from quit coach. The following services are considered nonstandard: meetings with quit coach in person, personalized emails, group chat online; information sent to family/friends.

b Significant at α = .05; because of rounding, some associations have 95% CIs that appear bounded by 1.00.

c Includes American Indian/Alaska Native, Asian/Asian American, Native Hawaiian/Pacific Islander, other, multiple races.

Smokers with more social support and greater nicotine dependence each had greater odds of expressing interest in multiple quitline services, primarily services that are nonstandard, such as a personalized web program or personalized text messages. In both bivariate and multivariable analyses, smokers who reported more frequent depressive symptoms had higher odds of interest in a mobile app (AOR, 1.08; 95% CI, 1.01–1.17) or personalized text messages (AOR, 1.11; 95% CI, 1.02–1.21) to help them quit (Table 4A).

Some participant characteristics were consistently associated with greater odds of being very interested in most or all quitline services in multivariable models. Smokers in the contemplation or preparation stage of readiness to quit had higher odds of reporting interest in each of 13 quitline services than smokers in the precontemplation stage. Black or African American smokers, compared with White smokers, had greater odds of being very interested in 12 of the 13 quitline services. Women had greater odds than men of being very interested in 8 of the 13 quitline services (Table 3, Table 4A, and Table 4B).

**Table 4B:** Multivariable Logistic Regression Results for Models Examining Associations Between Participant Characteristics and Interest in Nonstandard Quitline Services Among a Sample of Low-Income Adult Daily Smokers in 9 States (N = 1,605) Participating in a Telephone Survey on Quitline Services, 2020–2022^a^

Independent variables	Adjusted odds ratio (95% CI)			
	Meet quit coach in person (n = 1,339)	Personalized emails (n = 1,342)	Group chat online (n = 1,344)	Send information to family/friends (n = 1,344)
**Demographic**				
**Age group, y**				
≤40	1 [Reference]	1 [Reference]	1 [Reference]	1 [Reference]
41–60	1.31 (0.88–1.97)	1.24 (0.83–1.89)	0.96 (0.66–1.41)	0.86 (0.57–1.32)
≥61	1.51 (0.95–2.41)	1.24 (0.76–2.02)	0.85 (0.53–1.35)	1.03 (0.63–1.69)
**Sex**				
Male	1 [Reference]	1 [Reference]	1 [Reference]	1 [Reference]
Female	1.22 (0.89–1.69)	1.25 (0.89–1.76)	1.52 (1.09–2.17)^b^	1.26 (0.88–1.81)
**Race**				
Black or African American	2.31 (1.65–3.27)^b^	2.57 (1.80–3.70)^b^	1.63 (1.15–2.31)^b^	2.17 (1.49–3.17)^b^
White	1 [Reference]	1 [Reference]	1 [Reference]	1 [Reference]
Other^c^	1.67 (1.04–2.63)^b^	1.17 (0.69–1.94)	1.73 (1.09–2.70)^b^	1.08 (0.61–1.86)
**Annual pretax household income, $**				
<10,000	1 [Reference]	1 [Reference]	1 [Reference]	1 [Reference]
10,000–19,999	1.22 (0.88–1.70)	0.81 (0.57–1.17)	0.83 (0.58–1.17)	1.28 (0.89–1.85)
20,000	0.77 (0.52–1.14)	1.10 (0.74–1.62)	0.99 (0.67–1.45)	1.06 (0.69–1.62)
**Education**				
Less than high school	1 [Reference]	1 [Reference]	1 [Reference]	1 [Reference]
High school/GED	0.89 (0.62–1.28)	0.82 (0.56–1.19)	0.77 (0.53–1.12)	0.81 (0.55–1.19)
More than high school	1.04 (0.73–1.49)	0.92 (0.63–1.35)	0.94 (0.66–1.36)	0.60 (0.40–0.89)^b^
**Children aged <18 y living in home**				
No	1 [Reference]	1 [Reference]	1 [Reference]	1 [Reference]
Yes	0.81 (0.56–1.15)	0.74 (0.51–1.07)	1.05 (0.74–1.48)	0.67 (0.45–0.99)^b^
**Reside in rural zip code**				
No	1 [Reference]	1 [Reference]	1 [Reference]	1 [Reference]
Yes	1.27 (0.84–1.91)	1.55 (1.02–2.35)^b^	0.97 (0.62–1.48)	1.27 (0.80–1.98)
**Psychosocial factors**				
Perceived stress (scale 0–16)	1.00 (0.95–1.05)	1.02 (0.97–1.10)	0.99 (0.94–1.05)	1.03 (0.97–1.09)
Depressive symptoms (scale 0–6)	1.06 (0.97–1.16)	1.15 (1.05–1.26)^b^	1.06 (0.96–1.16)	1.04 (0.94–1.15)
Social support (scale 1–4)	1.07 (0.89–1.30)	1.21 (0.99–1.48)	1.18 (0.97–1.44)^b^	1.55 (1.24–1.95)^b^
**Tobacco-related factors**				
Nicotine dependence (scale 0–6)	1.20 (1.07–1.33)^b^	1.14 (1.02–1.27)^b^	1.06 (0.95–1.19)	1.03 (0.92–1.16)
Past use of cessation aids (vs no use)	1.29 (0.96–1.73)	1.31 (0.96–1.79)	1.51 (1.12–2.06)^b^	1.18 (0.85–1.64)
Ambivalence about smoking (scale 1–4)	1.21 (0.98–1.51)	1.19 (0.95–1.50)	1.23 (0.99–1.55)	1.10 (0.86–1.40)
Stage of readiness to quit				
Precontemplation	1 [Reference]	1 [Reference]	1 [Reference]	1 [Reference]
Contemplation	3.84 (2.26–7.01)^b^	4.01 (2.24–7.84)^b^	2.75 (1.63–4.93)^b^	5.53 (2.80–12.59)^b^
Preparation	4.61 (2.59–8.69)	5.83 (3.13–11.74)^b^	4.40 (2.51–8.14)^b^	7.47 (3.64–17.41)^b^

Abbreviation: GED, General Educational Development.

a The following services are considered standard quitline services: nicotine patches, gum, or lozenge; toll-free telephone number to quitline; printed quit smoking booklet; online lessons and exercises; telephone calls from quit coach. The following services are considered nonstandard: meetings with quit coach in person, personalized emails, group chat online; information sent to family/friends.

b Significant at α = .05; because of rounding, some associations have 95% CIs that appear bounded by 1.00.

c Includes American Indian/Alaska Native, Asian/Asian American, Native Hawaiian/Pacific Islander, other, multiple races.

## Discussion

In this study, low-income smokers reported their level of interest in 13 standard and nonstandard services currently available for free from at least some state tobacco quitlines. Participants tended to be most interested in services that are already standard (eg, NRT, the ability to call and speak with a counselor), but several nonstandard options also piqued their interest (eg, mobile phone apps, web-based programs).

Interest in quitline services varied in several ways. First, some participants were generally more interested than others, regardless of the type of service. For example, women and Black or African American participants were more interested in most services than were men and non–Black or African American participants. This finding aligns with prior studies showing higher enrollment of women in cessation services compared with men (21,22). Unsurprisingly, smokers in the contemplation and preparation stages also were more interested in nearly all services than those in the precontemplation stage. Nicotine dependence was associated with increased interest in nearly all services as well, which likely reflects well-known differences: smokers with greater nicotine dependence feel more pressure to quit, perceive quitting as more difficult, and have less confidence in quitting on their own compared with smokers with less nicotine dependence (23).

In other instances, however, interest in certain types of quitline services differed systematically by participant characteristics. For example, as participant age increased, so did interest in standard quitline services such as telephone counseling and printed materials. In contrast, as age decreased, interest grew in nonstandard services such as mobile apps or personalized web-based programs. These findings support our study hypotheses and suggest that age-based preferences for technology-mediated health behavior interventions may apply to smoking cessation in the same way demonstrated for other health behavior interventions (6–8).

Overall, up to two-thirds of low-income smokers reported being very interested or somewhat interested in receiving nonstandard quitline services such as a mobile app (65%), a personalized web program (59%), or personalized text messages (46%) to help them quit smoking. Evidence for the effectiveness of these interventions is growing. In 2020, the Community Preventive Services Task Force found strong evidence to recommend mobile telephone text messaging interventions for tobacco cessation and in 2019 found sufficient evidence to recommend internet-based interventions to do the same (24). Studies also suggest that adding digital interventions to cessation counseling programs improves effectiveness (25,26).

Digital interventions hold promise to reach low-income smokers and others unengaged by traditional intervention delivery formats (21). For example, texting interventions can be customized, are interactive, and provide users with on-demand tips and supports. Internet-based interventions can be interactive, can tailor messages for individuals or populations, and provide a wider range of content formats, such as text, images, and videos. However, low levels of participation in online services by racial and ethnic minority groups also has been documented and warrants further evaluation (22).

Of the 13 quitline services we examined, our sample of low-income, racially diverse smokers was most interested in receiving NRT, with 43% being very interested and another 29% somewhat interested. Meta-analyses of clinical trials support the efficacy of each of the 7 pharmacologic cessation aids approved by the US Food and Drug Administration, both alone and in combination (27). Cessation rates for counseling are improved when pharmacotherapy is added (28,29).

Perhaps most importantly, we found that on average, the low-income smokers in our sample were very interested in 3 or more services. Quitlines could design intervention packages or bundles to appeal to different kinds of smokers just as the tobacco industry adapts its products and marketing to targeted subgroups (30). Some quitlines have moved from offering standard packages to allowing smokers to choose the intervention(s) in which they have the most interest (31). Although findings from our study provide some initial hints about potential subgroups of smokers and the services they are interested in, additional research and audience segmentation analysis could further inform the design process.

Historically, campaigns to promote tobacco quitlines introduce reasons for smokers to quit, usually linking those reasons to a particular group. For example, campaigns are designed for pregnant women who smoke with messages about protecting their unborn child (32). But rarely have campaigns focused on types of services that are available and might uniquely appeal to certain subgroups. One exception is DEJELO YA, which in part sought to convey to Spanish-speaking smokers in New Mexico that quitline services were available in Spanish. During the campaign, calls from Spanish-speakers to the state tobacco quitline increased 31%, compared with an increase of only 3% among non-Hispanic people during the same time period (33). Future research should continue exploring the potential importance of providing service-related information in generating demand for and use of quitline services by different population groups.

### Limitations and strengths

Our study has several limitations. It was cross-sectional and we do not know which, if any, services participants actually used. Thus, we cannot determine whether being very interested in a particular quitline service translates into actually using the service or greater success quitting smoking. The eligibility criteria limited the representativeness of the sample. To be included in the study, participants had to reach out to 2-1-1 in their community, and only those who allowed smoking in their homes and smoked daily were eligible for the study. Less than half (47%) of those reached by the study team enrolled in the study and completed a baseline interview. Because we collected no data from those who were ineligible or declined participation, we cannot assess how they may have differed from those who enrolled. However, the study was successful in enrolling the desired population of low-income smokers. Because these analyses were exploratory, we did not adjust for multiple comparisons, and some associations may have been due to chance.

These potential limitations are offset to some extent by strengths of the study, which include a multistate sample with much lower levels of income than reported in other surveillance data of smoking prevalence in the US (34), and inclusion of smokers not currently thinking about quitting. The latter group often is not represented in tobacco quitline research, and the findings demonstrate that their inclusion suppresses the level of interest in quitline services reported in the overall sample.

### Conclusion

Low-income smokers from across the US expressed interest in a range of nonstandard services that could be provided by state tobacco quitlines to reach and engage a larger number of low-income smokers. Preferences for specific types of services sometimes varied by smoker characteristics. Younger smokers were more interested than older smokers in digital and online cessation services, as were women and smokers with greater nicotine dependence.

## Appendix Supplemental Tables

**Appendix:** Supplemental Table 1. Bivariate Associations Between Participant Characteristics and Interest in Standard Tobacco Quitline Services Among a Sample of Low-Income Adult Daily Smokers in 9 States (N = 1,605) Participating in a Telephone Survey on Quitline Services, 2020–2022

Independent variables	Odds ratio (95% CI)				
	Nicotine patches, gum, or lozenges	Toll–free telephone number to quitline	Printed quit smoking booklet	Online lessons and exercises	Telephone calls from quit coach
**Demographic characteristics**					
**Age group, y**					
21–40	1 [Reference]	1 [Reference]	1 [Reference]	1 [Reference]	1 [Reference]
41–60	1.37 (1.07–1.78)^a^	1.58 (1.20–2.09)^a^	1.81 (1.33–2.48)^a^	1.18 (0.87–1.59)	1.59 (1.13–2.29)^a^
≥61	1.19 (0.89–1.60)	1.81 (1.32–2.48)^a^	2.13 (1.52–3.03)^a^	1.07 (0.76–1.53)	1.46 (0.97–2.20)
**Female (vs male)**	1.27 (1.03–1.57)^a^	1.43 (1.14–1.81)^a^	1.59 (1.24–2.05)^a^	1.57 (1.20–2.06)^a^	1.43 (1.07–1.92)^a^
**Race**					
Black or African American	1.06 (0.86–1.31)	1.30 (1.04–1.63)^a^	1.65 (1.29–2.10)^a^	1.66 (1.29–2.15)^a^	2.01 (1.51–2.69)
White	1 [Reference]	1 [Reference]	1 [Reference]	1 [Reference]	1 [Reference]
Other	1.09 (0.79–1.51)	1.00 (0.70–1.41)	1.82 (1.28–2.58)^a^	1.82 (1.25–2.62)^a^	2.07 (1.37–3.11)^a^
**Hispanic ethnicity**	1.03 (0.64–1.65)	0.92 (0.54–1.50)	1.37 (0.82–2.23)	1.14 (0.64–1.93)	1.15 (0.61–2.03)
**Annual pretax household income, $**					
<10,000	1 [Reference]	1 [Reference]	1 [Reference]	1 [Reference]	1 [Reference]
10,000–19,999	1.14 (0.90–1.46)	1.01 (0.79–1.30)	1.13 (0.87–1.47)	1.18 (0.69–1.57)	0.84 (0.61–1.15)
≥20,000	0.97 (0.75–1.25)	0.81 (0.62–1.06)	0.80 (0.60–1.07)	0.94 (0.69–1.28)	0.77 (0.55–1.06)
**Education**					
Less than high school	1 [Reference]	1 [Reference]	1 [Reference]	1 [Reference]	1 [Reference]
High school/GED	0.92 (0.71–1.19)	1.05 (0.80–1.37)	0.78 (0.58–1.03)	0.75 (0.55–1.02)	0.80 (0.57–1.12)
More than high school	0.98 (0.76–1.25)	1.05 (0.81–1.37)	0.87 (0.67–1.15)	0.99 (0.75–1.33)	0.89 (0.65–1.23)
**Children <18 y living in home (vs none)**	0.95 (0.75–1.19)	0.79 (0.62–1.01)	0.87 (0.67–1.12)	0.93 (0.70–1.22)	0.96 (0.71–1.30)
**Reside in rural zip code (vs nonrural)**	1.04 (0.79–1.37)	1.16 (0.87–1.53)	1.13 (0.83–1.52)	1.02 (0.73–1.39)	0.62 (0.41–0.92)
**Psychosocial factors**					
Perceived stress (scale 0–16)	1.01 (0.98–1.04)	1.01 (0.98–1.05)	0.98 (0.95–1.01)	1.02 (0.99–1.06)	1.01 (0.97–1.05)
Depressive symptoms (0–6)	1.06 (1.00–1.11)^a^	1.08 (1.02–1.14)^a^	1.03 (0.97–1.10)	1.03 (0.97–1.10)	1.05 (0.98–1.13)
Social support (1–4)	0.97 (0.87–1.10)	1.10 (0.96–1.26)	1.36 (1.17–1.58)^a^	1.30 (1.11–1.52)^a^	1.00 (0.86–1.19)
**Tobacco–related factors**					
Nicotine dependence smoking (scale 0–6)	1.08 (1.00–1.16)^a^	1.01 (0.94–1.09)	1.00 (0.92–1.09)	1.05 (0.96–1.14)	1.06 (0.97–1.17)
Past use of cessation aids (vs. no use)	1.81 (1.48–2.21)^a^	1.65 (1.34–2.05)^a^	1.51 (1.20–1.90)^a^	1.42 (1.12–1.81)^a^	1.73 (1.32–2.28)^a^
Live with other smokers (vs. not)	0.88 (0.72–1.07)	0.85 (0.69–1.05)	0.84 (0.67–1.05)	0.97 (0.76–1.23)	0.81 (0.62–1.06)
Ambivalence about smoking (scale 1–4)	1.58 (1.35–1.85)^a^	1.73 (1.46–2.04)^a^	1.46 (1.23–1.74)^a^	1.51 (1.25–1.82)^a^	1.57 (1.28–1.95)^a^
Stage of readiness to quit					
Precontemplation	1 [Reference]	1 [Reference]	1 [Reference]	1 [Reference]	1 [Reference]
Contemplation	4.81 (3.47–6.79)^a^	5.86 (3.95–9.03)^a^	4.17 (2.77–6.54)^a^	4.49 (2.85–7.47)^a^	7.35 (3.92–15.68)^a^
Action	5.74 (3.98–8.41)^a^	8.37 (5.45–13.25)^a^	6.91 (4.44–11.11)^a^	7.08 (4.36–12.05)^a^	12.37 (6.45–26.82)^a^

Abbreviation: GED, General Educational Development.

^a^ Significant at α = .05.

**Appendix:** Supplemental Table 2A. Bivariate Associations Between Participant Characteristics and Interest in Nonstandard Tobacco Quitline Services^a^ Among a Sample of Low–Income Adult Daily Smokers in 9 States (N = 1,605) Participating in a Telephone Survey on Quitline Services, 2020–2022

Independent variables	Odds ratio (95% CI)			
	Mobile app	Personalized web program	Personalized text messages	Chat online with quit coach
**Demographic characteristics**				
**Age group, y**				
21–40	1 [Reference]	1 [Reference]	1 [Reference]	1 [Reference]
41–60	0.76 (0.59–0.99)^b^	0.98 (0.75–1.29)^b^	1.39 (1.02–1.91)^b^	1.23 (0.89–1.70)
≥61	0.65 (0.48–0.88)^b^	0.74 (0.53–1.03)^b^	1.27 (0.89–1.83)	1.36 (0.95–1.97)
**Female (vs male)**	1.64 (1.31–2.08)^b^	1.49 (1.17–1.92)^b^	1.49 (1.14–1.96)^b^	1.97 (1.48–2.66)^b^
**Race**				
Black or African American	1.15 (0.92–1.43)	1.37 (1.08–1.74)^b^	1.58 (1.23–2.04)	1.37 (1.06–1.79)^b^
White	1 [Reference]	1 [Reference]	1 [Reference]	1 [Reference]
Other	0.99 (0.70–1.39)	1.12 (0.77–1.61)	1.11 (0.74–1.66)	1.09 (0.72–1.63)
**Hispanic ethnicity**	1.14 (0.70–1.86)	0.96 (0.55–1.61)	0.92 (0.50–1.60)	0.94 (0.50–1.66)
**Annual pretax household income, $**				
<10,000	1 [Reference]	1 [Reference]	1 [Reference]	1 [Reference]
10,000–19,999	1.09 (0.84–1.40)	1.26 (0.96–1.65)	0.79 (0.59–1.06)	1.01 (0.76–1.37)
≥20,000	1.17 (0.90–1.52)	1.17 (0.88–1.55)	0.79 (0.59–1.07)	0.94 (0.69–1.29)
**Education**				
Less than high school	1 [Reference]	1 [Reference]	1 [Reference]	1 [Reference]
High school/GED	0.83 (0.63–1.08)	0.97 (0.72–1.29)	0.66 (0.48–0.89)^b^	0.75 (0.54–1.03)
More than high school	1.09 (0.84–1.41)	1.18 (0.89–1.55)	0.78 (0.59–1.04)	0.98 (0.73–1.32)
**Children <18 y living in home (vs none)**	1.16 (0.92–1.47)	0.93 (0.71–1.20)	0.98 (0.74–1.29)	1.10 (0.83–1.45)
**Reside in rural zip code (vs nonrural)**	0.89 (0.66–1.19)	0.84 (0.61–1.14)	0.96 (0.69–1.33)	1.03 (0.73–1.44)
**Psychosocial factors**				
Perceived stress (scale 0–16)	1.05 (1.01–1.08)^b^	1.03 (0.99–1.06)	1.00 (0.97–1.04)	1.03 (0.99–1.06)
Depressive symptoms (scale 0–6)	1.11 (1.05–1.17)^b^	1.07 (1.01–1.14)	1.08 (0.02–1.16)^b^	1.09 (1.02–1.16)^b^
Social support (scale 1–4)	1.11 (0.97–1.27)	1.18 (1.02–1.37)^b^	1.24 (1.06–1.45)^b^	1.18 (1.00–1.38)^b^
**Tobacco–related factors**				
Nicotine dependence (scale 0–6)	1.07 (0.99–1.15)	1.09 (1.00–1.18)^b^	1.10 (1.01–1.20)^b^	1.11 (1.01–1.21)^b^
Past use of cessation aids (vs no use)	1.61 (1.31–1.99)^b^	1.54 (1.23–1.93)^b^	1.70 (1.34–2.18)^b^	1.68 (1.30–2.16)^b^
Live with other smokers (vs not)	1.12 (0.91–1.38)	0.95 (0.76–1.19)	1.07 (0.84–1.36)	1.17 (0.91–1.50)
Ambivalence about smoking (scale 1–4)	1.53 (1.31–1.81)^b^	1.43 (1.21–1.71)^b^	1.45 (1.20–1.75)^b^	1.70 (1.40–2.08)^b^
Stage of readiness to quit				
Precontemplation	1 [Reference]	1 [Reference]	1 [Reference]	1 [Reference]
Contemplation	4.18 (2.95–6.06)^b^	6.18 (3.94–10.24)^b^	5.04 (3.14–8.60)^b^	6.83 (3.99–12.76)^b^
Action	4.59 (3.12–6.89)^b^	8.05 (4.97–13.68)^b^	6.93 (4.18–12.11)^b^	8.24 (4.66–15.76)^b^

Abbreviation: GED, General Educational Development.

^a^ The following services are considered standard quitline services: nicotine patches, gum, or lozenge; toll–free telephone number to quitline; printed quit smoking booklet; online lessons and exercises; telephone calls from quit coach.

^b^ Significant at α = .05.

**Appendix:** Supplemental Table 2B. Bivariate Associations Between Participant Characteristics and Interest in Nonstandard Tobacco Quitline Services^a^ Among a Sample of Low–Income Adult Daily Smokers in 9 States (N = 1,605) Participating in a Telephone Survey on Quitline Services, 2020–2022

Independent variables	Odds ratio (95% CI)			
	Meet quit coach in person	Personalized emails	Group chat online	Send info to family/friends
**Demographic characteristics**				
**Age group, y**				
21–40	1 [Reference]	1 [Reference]	1 [Reference]	1 [Reference]
41–60	1.52 (1.09–2.15)^b^	1.57 (1.10–2.27)^b^	1.03 (0.75–1.43)	1.15 (0.80–1.69)
≥61	1.84 (1.26–2.70)^b^	1.68 (1.12–2.53)^b^	0.99 (0.68–1.45)	1.42 (0.94–2.16)
**Female (vs male)**	1.25 (0.95–1.64)	1.31 (0.98–1.77)	1.61 (1.20–2.17)^b^	1.19 (0.87–1.63)
**Race**				
Black or African American	1.77 (1.35–2.31)^b^	1.90 (1.43–2.54)^b^	1.52 (1.15–2.01)^b^	1.90 (1.40–2.58)^b^
White	1 [Reference]	1 [Reference]	1 [Reference]	1 [Reference]
Other	1.60 (1.06–2.37)^b^	1.34 (0.85–2.07)	1.92 (1.29–2.84)^b^	1.17 (0.70–1.89)
**Hispanic ethnicity**	1.16 (0.63–2.00)	0.97 (0.49–1.76)	1.10 (0.59–1.95)	1.48 (0.78–2.61)
**Annual pretax household income, $**				
<10,000	1 [Reference]	1 [Reference]	1 [Reference]	1 [Reference]
10,000–19,999	1.08 (0.81–1.45)	0.75 (0.54–1.03)	0.83 (0.61–1.13)	1.14 (0.82–1.59)
≥20,000	0.65 (0.46–0.90)^b^	0.81 (0.58–1.12)	0.90 (0.65–1.24)	0.79 (0.54–1.13)
**Education**				
Less than high school	1 [Reference]	1 [Reference]	1 [Reference]	1 [Reference]
High school/GED	0.79 (0.58–1.09)	0.67 (0.48–0.94)^b^	0.70 (0.50–0.97)^b^	0.74 (0.52–1.04)
More than high school	0.91 (0.67–1.23)	0.84 (0.61–1.15)	0.96 (0.71–1.32)	0.57 (0.41–0.81)^b^
**Children <18 y living in home (vs none)**	0.84 (0.62–1.12)	0.70 (0.50–0.96)^b^	1.20 (0.90–1.60)	0.79 (0.56–1.11)
**Reside in rural zip code (vs nonrural)**	0.99 (0.69–1.38)	1.04 (0.72–1.48)	0.84 (0.58–1.21)	1.00 (0.67–1.46)
**Psychosocial factors**				
Perceived stress (scale 0–16)	1.01 (0.97–1.04)	1.04 (1.00–1.08)	1.00 (0.97–1.04)	1.00 (0.96–1.04)
Depressive symptoms (scale 0–6)	1.08 (1.01–1.16)^b^	1.12 (1.05–1.21)^b^	1.06 (0.99–1.14)	1.01 (0.93–1.09)
Social support (scale 1–4)	1.04 (0.89–1.22)	1.13 (0.96–1.35)	1.20 (1.01–1.42)^b^	1.43 (1.18–1.74)^b^
**Tobacco–related factors**				
Heaviness of smoking (scale 0–6)	1.09 (1.00–1.21)^b^	1.07 (0.97–1.18)	1.06 (0.96–1.16)	0.99 (0.89–1.09)
Past use of cessation aids (vs no use)	1.55 (1.20–2.00)^b^	1.55 (1.18–2.03)^b^	1.65 (1.26–2.16)^b^	1.42 (1.06–1.90)^b^
Live with other smokers (vs not)	0.93 (0.73–1.20)	0.97 (0.74–1.26)	0.93 (0.71–1.21)	1.27 (0.96–1.69)
Ambivalence about smoking (scale 1–4)	1.46 (1.20–1.78)^b^	1.46 (1.19–1.80)^b^	1.48 (1.21–1.81)^b^	1.35 (1.09–1.68)^b^
Stage of readiness to quit				
Precontemplation	1 [Reference]	1 [Reference]	1 [Reference]	1 [Reference]
Contemplation	4.63 (2.85–8.02)^b^	5.47 (3.13–10.53)^b^	3.06 (1.95–5.05)^b^	6.75 (3.49–15.15)^b^
Action	6.19 (3.68–10.99)^b^	8.09 (4.49–15.89)^b^	4.60 (2.83–7.80)^b^	10.21 (5.13–23.32)^b^

Abbreviation: GED, General Educational Development.

^a^ The following services are considered standard quitline services: nicotine patches, gum, or lozenge; toll–free telephone number to quitline; printed quit smoking booklet; online lessons and exercises; telephone calls from quit coach.

^b^ Significant at α = .05.
